# Differential Profile of Specialist Aggressor versus Generalist Aggressor in Child-to-Parent Violence

**DOI:** 10.3390/ijerph19095720

**Published:** 2022-05-08

**Authors:** María J. Navas-Martínez, M. Carmen Cano-Lozano

**Affiliations:** Department of Psychology, University of Jaén, 23071 Jaén, Spain; mccano@ujaen.es

**Keywords:** adolescents, specialist aggressors, generalist aggressors, violence profiles, child-to-parent violence

## Abstract

Research on violence in general highlights the need to differentiate between those aggressors who only show specialized violence in the family context and those who also show generalized violence in other contexts outside the family. However, in the phenomenon of child-to-parent violence (CPV), the distinctive characteristics of this profile have not been yet analyzed. The aim of this study was to identify the typology of specialist aggressor versus the typology of generalist aggressor and examine whether they differ in their characteristics. A total of 1559 CPV aggressors participated, with ages between 12 and 18 years, of whom 22.4% exerted violence only towards parents (specialist aggressors) and 77.6% also exerted violence towards peers (generalist aggressors). The results show that specialized violence and generalized violence seem to follow different patterns according to age. The generalists were characterized by a more negative profile than the specialists. Specifically, the former showed more CPV and for more reasons, both reactive and proactive. Regarding individual characteristics, they obtained lower levels of emotional intelligence and resilience. Concerning family characteristics, they presented higher levels of insecure parental attachment and parental violence (direct and observed). The predictive variables retained in the regression model represented approximately 16.4% of the variation in the type of aggressor. This study supports the classification based on the specificity versus generality of violence, as it was found that specialist and generalist CPV aggressors differ significantly in their characteristics. It is considered that the findings could help to identify the differential mechanisms through which both types of aggressors have developed CPV. Further analysis of this profile can be of great use for the design of intervention and prevention programs adapted to the needs of each typology.

## 1. Introduction

In the last few decades, studies carried out in different countries suggest that a considerable proportion of adolescents exert some type of violence towards their parents (child-to-parent violence, hereinafter CPV). Specifically, in community samples, the frequency of psychological violence ranges between 28.8% and 91.5% [1,2,3,4,5,6], that of physical violence ranges between 2.5% and 25% [1,2,3,4,5,6], that of financial violence ranges between 9.3% and 50.9% [2,3,5,6], and control and domain behaviors towards the parents are around 70% [2]. In judicial samples, for example in Spain, the rates show an upward trend in CPV offenses since the year 2005, reaching a total of 5055 initiated procedures for this offense in the year 2019 [7]. The possible reasons for the increase in this type of violence include social changes related to cultural values, parenting methods and social interaction. In particular, CPV refers to those behaviors that cause psychological, physical and/or financial damage carried out by a son or daughter towards a father or mother [8] intentionally and repeatedly in time [9]. Violent behaviors that may occur sporadically or as a result of psychological or developmental disorders are excluded [10] since the objective of this type of violence is to obtain control and domain over the parents or the people that play their role [11].

Most studies on CPV have been focused on analyzing the individual, family and social characteristics of aggressors of this type of violence (see the review of Simmons et al. [12]). However, few studies have explored if there are different typologies or profiles of aggressors towards the parents [13,14]. The results of these studies, together with those found in studies on other types of violence [15,16,17,18,19] suggest that analyzing the heterogeneity of aggressors can improve the understanding of not only their common characteristics but also, and most importantly, the characteristics that differentiate them. Such understanding can provide the keys to proposing different etiological mechanisms of the violent behavior, which, in turn, could have a positive impact on the efficacy of the interventions designed from such differential mechanisms. Therefore, the aim of this study was to explore a CPV aggressor profile that has not been analyzed to date. In particular, the study identifies the typology of the aggressor who shows violence towards parents (specialist) and the typology of the aggressor who shows violence towards both parents and peers (generalist) and examines whether they present differential characteristics.

There are two main categories traditionally proposed to classify different types of aggressors [20,21]. The first category, which includes the severity and frequency of the violence, provides three subtypes of aggressors depending on whether they show violent behavior of low, moderate or high severity and frequency. The second category, which includes the versatility or generality of the violence, provides two subtypes of aggressors depending on whether the violence is shown specifically in the family context (specialist aggressor) or it is generalized also towards other contexts outside the family (generalist aggressor). It has been suggested that these two classifications could be related, since generalist aggressors tend to show more severe and frequent violence than specialist aggressors [22]. Likewise, another classification based on the experience of victimization [23] allows distinguishing between aggressors with and without a history of experienced violence. Currently, these classifications have empirical support regarding the establishment of different aggressor profiles. For example, in CPV, the differences between aggressors have been analyzed mostly as a function of the severity of the violence exerted [24]; in gender-based violence, they mostly as a function of the versatility of the violence exerted [16,20,22,25]; and in violence towards peers, they mostly as a function of the victimization or violence experienced [26,27].

Coghlan and Millsteed [28] highlighted that understanding different profiles of CPV aggressors has important implications for the development and implementation of effective intervention programs. Reaching such understanding involves two tasks. Firstly, it is necessary to identify the different types of CPV aggressors. To our knowledge, only two studies have addressed this topic, providing, in a community population, the typology of the victimized aggressor [14] and, in a judicial population, the typology of the aggressor specialized in CPV offense versus the aggressor who engages in CPV offense as well as in other types of offenses [13]. Following the differentiation proposed by Kuay et al. [29] in their trait-based model of CPV, this typology is related to the specialist aggressor and the generalist aggressor. Secondly, it is necessary to analyze the differential characteristics of the different typologies. In this sense, while the profile of victimized aggressor has already been analyzed [14], the differences between specialists and generalists have only been studied in a judicial sample and only in relation to demographic variables [13]. However, according to the theoretical approach of Kuay et al. [29], specialists and generalists differ in other variables. More specifically, generalists are characterized by high levels of emotional insensibility traits, as well as by permissive parental discipline. On the other hand, specialists present low levels of emotional insensibility traits and strict parental discipline. Empirical studies have found that the gender of the minors with CPV offenses differs significantly between specialists and generalists, with a greater proportion of male generalists, whereas the age of the aggressors does not show differences [13]. In the field of gender-based violence, there are significant differences in the age of men classified as specialist and generalist aggressors, with the latter being younger [16,17]. In gender-based violence, several authors have also explored, in clinical and judicial populations, the differences among these aggressors in some individual and family characteristics, such as psychopathology, drug use and family violence [16,20]. However, in CPV, it is still unknown whether both types of aggressors differ in their characteristics.

The lack of research on this topic has some consequences, for example, in the design of interventions for CPV. A recent systematic review identified that, currently, there are six intervention programs that work on this type of violence, half of which are applied in the judicial context (see the review of Toole-Anstey et al. [30]). Since traditional research has not differentiated between specialist and generalist aggressors [28], none of these programs contemplate a differential treatment for minors who have exclusively committed CPV offenses and for those who have committed other violent offenses in addition to CPV. However, Grace-Moulds et al. [13], in a sample of 305 offenders, observed that 14.75% exclusively committed CPV offenses, whereas the other 85.25% committed other violent and non-violent offenses in addition to CPV; therefore, they concluded that most CPV offenses occur as a part of a broader or “generalized” pattern of antisocial behavior. In this sense, although both types of aggressors may have common characteristics, it is also possible that they have specific and distinctive characteristics. Following this approach, the current interventions could be covering some of the needs of the aggressors, but not all of them. Knowing these aspects could improve the efficacy of the current interventions if they are designed taking into account the common and differential characteristics of both types of aggressors, which would justify the need to analyze this profile.

In the context of research on CPV, the typology of the specialist aggressor refers to the adolescent who exerts violent behaviors aimed specifically towards his/her parents and does not exert violence towards other people. On the other hand, the generalist aggressor refers to the adolescent who exerts violent behaviors towards his/her parents and also towards other people [29]. The type of juvenile violence that this study includes in the category of generalist aggressor is violence towards peers (bullying and/or cyberbullying) for two fundamental reasons: (1) violence towards peers has been consistently related to CPV, and (2) both types of violence share many of the characteristics analyzed in this study. The literature shows that violence towards peers is one of the types of juvenile violence that have been most frequently related to CPV [31,32,33], and it also reports that violence towards peers is a significant predictor of CPV [31,33]. In fact, recent studies show that adolescents who exert high levels of violence towards parents also exert high levels of violence towards peers [34]. Despite these evidences, studies have analyzed both types of juvenile violence independently, without considering the possibility that a large proportion of CPV aggressors also exert violence towards peers.

With respect to the pattern of violence of both types of aggressors, in other fields of research, it has been found that generalists exert more violence than specialists towards other people [22] and towards their parents [20]. In CPV, 75% of generalists have committed violent offenses, whereas the other 25% have committed non-violent offenses [13], which suggests that most of these aggressors show high levels of violence. However, these studies did not examine the pattern of violence of specialists, and they did not analyze the possible differences from the pattern of violence of generalists. In this line, it would be interesting to know whether both types differ in the levels of violence exerted towards the parents, as well as in the type of violence. Regarding the reasons for the violence, two types have been identified: reactive (or defensive) and proactive (or instrumental) [35]. When the violence takes place as a response to a perceived threat, there are reactive reasons, whereas when the violence is the instrument used to obtain personal benefits, there are proactive reasons. According to the theoretical model of Kuay et al. [29], specialist aggressors tend to show more reactive violence towards their parents in response to strict parental discipline, whereas, in generalist aggressors, the violence tends to be more proactive, as a consequence of permissive discipline.

With regard to the characteristics of the aggressors, there are currently numerous individual and family variables that have been associated with CPV [14,36,37,38,39,40,41,42]. However, many of these variables have also been linked to the violence exerted towards peers [26,43,44,45,46,47,48]. Thus, although the literature has identified different characteristics associated with the development of CPV, it also suggests that at least some of them would not be specific to this type of violence and would be present in other types of violence. Concerning the individual characteristics, emotional intelligence has been negatively related to CPV [38,40,42] and violence towards peers [26,44]. Moreover, difficulty in identifying emotions has been pointed out as a significant predictor of CPV [42] and violence in general [45]. Furthermore, low resilience and difficulty in adapting to conflicting and adverse situations [49] have also been related to CPV [14] and violence towards peers [47].

Regarding family characteristics, parental attachment [50] is the cognitive representation that the child builds with his/her parents. Through interaction, an emotional bond is generated, which is modified over time depending on the experiences of attachment [51]. Children develop secure attachment bonds if their main role models provide them with security and affection. When these elements fail, an insecure bond is developed, resulting in preoccupied, avoidant or traumatized attachment styles. This variable has also been associated with CPV [14,39,41] and with violence towards peers [46,48]. Moreover, it has been found that some elements of insecure attachment, such as the lack of parental warmth perceived by the adolescent, are related to psychological and social difficulties that lead to CPV [36]. The quality of attachment is greatly influenced by the family context [52]. The importance of the context (e.g., parental ineffectiveness) and the mode (e.g., parental warmth) with which parents discipline their children for CPV has been highlighted [53]. However, exposure to violence in the family context (or exposure to parental violence) has been pointed out as one of the best predictors of the development of CPV and also of violence towards peers [6,31,37,43,54,55,56,57,58]. This evidence is mainly based on the theoretical presumptions of social learning [59], which assume that children reproduce the behavioral pattern that they are exposed to. Children who suffer violence from their parents or observe violence between their parents tend to internalize and learn such behavioral patterns, and then they reproduce them, using violence to solve conflicts with their parents, in a bidirectional dynamic of violence [60], and with other social agents, such as peers.

As has been shown, only one study on CPV has addressed the differences between specialists and generalists in a judicial sample, and only in relation to sociodemographic variables, such as gender and age. However, the findings of the mentioned study could only be generalized to the judicial context. This can involve an added limitation in the understanding of this profile, since this sample is representative only of the most serious cases of CPV, i.e., those that are reported to the police [61]. In this sense, it would be of interest to examine the differences between the two types of aggressors in community samples and in relation to other variables usually associated with CPV. 

In view of the above-mentioned research, the aim of this study was to explore whether there are differences between two types of CPV aggressors: specialists (those who have shown violent behavior only towards parents) and generalists (those who have shown violent behavior towards parents and peers). To determine whether these two types of aggressors are different, two research questions were suggested: (1) Do aggressors belong to the same or different groups? (2) Do they share the same or different characteristics? [18]. Following the procedure used in the mentioned study, this work analyzed, firstly, how aggressors are distributed in both groups, to verify whether there is a considerable number of members per group. In such a case, there would be evidence of the existence of these two types of aggressors. It is expected that each group will have a considerable number of members and a greater proportion of generalist aggressors than specialist aggressors [13]. Secondly, the study analyzed the differences between the two groups in a set of variables that have been consistently linked to CPV and to violence towards peers. The detection of statistically significant differences in a considerable number of variables would indicate the existence of two differential aggressor profiles. Specifically, we analyzed the differences between specialist aggressors and generalist aggressors in their sociodemographic characteristics (gender and age), pattern of CPV (types and reasons for CPV) and individual (emotional intelligence and resilience) and family characteristics (parental attachment style, family functioning, and exposure to parental violence). We also explored the contribution of these variables in the prediction of the type of aggressor. As has been reported in similar studies [20,25], it is expected that both types of aggressors differ in the examined variables.

## 2. Materials and Methods

### 2.1. Participants

From a total of 3142 adolescents from two provinces of southern Spain, we selected those who had shown repeated behaviors of CPV (any behavior exercised 2 or more times) in the last year. The final sample consisted of 1559 aggressors (54.6% females), aged between 12 and 18 years (*M*_age_ = 14.5, *SD* = 1.5), from charter (50.5%) and public (49.5%) educational centers. Following the most common classification method used in similar studies [14,20,25], the participants were divided into two groups based on self-reported violence: the group of generalist aggressors (77.6%, *n* = 1210) and the group of specialist aggressors (22.4%, *n* = 349).

### 2.2. Tools

The *Child-to-Parent Violence Questionnaire, Adolescents Version* (CPV*-Q-A* [62]), evaluates the frequency of behaviors of psychological, physical, financial and control/domain violence exerted towards the mother (α = 0.67) and the father (α = 0.66) in the last year through 14 parallel items scored on a Likert scale (0 = *never*; 4 = *very often*, 6 *times or* *more*). An example is “I have demanded my parents that at home they have to do what I want”. The second part of the questionnaire assessed the frequency of the reasons for exerting violence towards the mother (reactive: α = 0.61; proactive: α = 0.66) and the father (reactive: α = 0.59; proactive: α = 0.66) through 8 parallel items scored on a Likert scale (0 = *never*; 3 = *always*). An example is “In response to a previous physical aggression from your father/mother (e.g., slap, punch, shove…)”.

The *European Bullying/Cyberbullying Intervention Project Questionnaires* (*EBIP-Q* and *ECIP-Q* [63], Spanish validation [64]) evaluate the frequency of violent behaviors exerted towards peers at school and/or in the street (bullying) and through digital media (cyberbullying) in the last two months through 7 items (α = 0.82) and 11 items (α = 0.75), respectively, scored on a Likert scale (0 = *no*; 4 = *yes, more than once a week*). An example is “I have excluded or ignored someone”.

The *Wong–Law Emotional Intelligence Scale* (*WLEIS* [65], Spanish validation [66]) evaluates four dimensions of emotional intelligence (assessment and expression of one’s own emotions, assessment and recognition of other people’s emotions, use or assimilation of one’s own emotions for personal performance, and regulation of one’s own emotions) through 16 items (α = 0.84) scored on a Likert scale (1 = *completely disagree*; 7 = *completely agree*). An example is “I am able to control my temper so that I can handle difficulties rationally”.

The *Connor–Davidson Resilience Scale, Short-Version* (*CD-RISC-10* [67], Spanish validation [68]), evaluates the degree of resilience to or coping with conflicts through 10 items (α = 0.79) scored on a Likert scale (degree of agreement: 0 = *not at all*; 4 = *almost always*). An example is “I believe that I am a strong person when faced with life’s challenges and difficulties”.

The *Attachment Representation Questionnaire, Short Version* (*CAMIR-R* [69], Spanish validation [70]), evaluates the experiences of parental attachment, determining a secure or insecure (preoccupied, avoidant and traumatized) attachment style, as well as the family functioning (parental permissiveness and value granted to parental authority), through 32 items (α = 0.65) scored on a Likert scale (1 = *strongly disagree*; 5 = *strongly agree*). An example is “From my experience as a child I have come to understand that we are never good enough for parents”.

The *Violence Exposure Scale* (*VES* [54]) evaluates the frequency with which the adolescent has experienced behaviors of psychological, physical and verbal violence from parents (direct parental violence) and the frequency with which the adolescent has observed behaviors of psychological, physical and verbal violence between parents (observed parental violence) through 6 items (α = 0.87) and 3 items (α = 0.73), respectively, scored on a Likert scale (0 = *never*; 4 = *every day*). An example is “How many times did your parents insult or humiliate you?”.

### 2.3. Procedure

Following the classification proposed by Montero and León [71], this study presents a descriptive, survey-based, cross-sectional design. The criteria for belonging to the “generalist” category were: exerting bullying (any behavior 1 or more times) or exerting cyberbullying (any behavior 1 or more times) in the last two months. On the other hand, the criteria for belonging to the “specialist” category were: exerting neither bullying (any behavior 0 times) nor cyberbullying (any behavior 0 times). The research was conducted in accordance with the Declaration of Helsinki. Firstly, it was authorized by the Ethics Committee of the University of Jaén (protocol number MAR.18/5.PRY). Then, it was authorized by the public administrations in the field of education and by each educational center. The parents and adolescents were informed about the study, and their signed informed consent to participate was obtained. The participation of the adolescents consisted of completing a set of voluntarily, anonymous and confidential paper-and-pencil questionnaires administered by a single evaluator in person and in groups in the educational centers.

### 2.4. Data Analysis

Firstly, we carried out contingency analyses with χ^2^ comparisons to determine the differences in the distribution of the aggressors as a function of gender and age.

Then, we analyzed the differences between generalist and specialist aggressors in the study variables, and the effect size was calculated for the intergroup differences with Cohen’s *d* statistic test [72]. Specifically, we analyzed whether the two types of aggressors differ in the pattern of CPV (types and reasons for CPV), their individual characteristics (emotional intelligence and resilience) and their family characteristics (parental attachment style, family functioning and exposure to parental violence). The data did not meet the assumptions of normality and homoscedasticity; thus, we applied the Mann–Whitney *U* test, which was designed to study the differences between groups with different distributions and variability.

A logistic regression analysis was also performed to explore the contribution of the analyzed variables in the prediction of the type of aggressor. The values 0 and 1 were assigned to the specialist and generalist aggressors, respectively. The significance of the parameters of the model was verified through Wald’s test, with a significance level of α = 0.05.

## 3. Results

Regarding the sociodemographic characteristics, there were no gender differences between the generalists and the specialists (χ*^2^* (1, 1558) = 0.06, *p* = 0.799) (see Table 1). On the other hand, differences were found in the proportions of aggressors distributed in the different age groups (χ*^2^* (2, 1557) = 6.9, *p* = 0.031). In general, the results show a greater proportion of generalists (64.8%) than specialists (57.2%) around the central ages of adolescence (14–16 years) and a greater proportion of specialists (42.9%) than generalists (35.2%) in both ends of this evolutionary stage (12–13 years and 17–18 years).

Statistically significant differences were found between the two types of aggressors in CPV and in the reasons for exerting it (see Table 2). Specifically, the generalist aggressors obtained higher scores in the CPV exerted towards the mothers and the fathers. Apart from being significant, these differences are especially relevant since they count medium effect sizes (CPV mother *d* = 0.60; CPV father *d =* 0.54). More specifically, except in control/domain violence towards the father, the generalists obtained higher scores in all the types of CPV compared to the specialists, with medium effect sizes (psychological CPV) and small but acceptable effect sizes (physical, financial and control/domain CPV). Regarding the reasons, the results show that the generalists obtained higher scores in the reactive and proactive reasons for exerting violence towards both the mothers and the fathers. The magnitude of the differences was small and medium for the reactive and proactive reasons, respectively.

Statistically significant differences were also found between the two types of aggressors in the individual variables, with relevant effect sizes (see Table 3). Specifically, the generalist aggressors obtained lower scores in the perception of their own emotions, in the assimilation of their own emotions and in the regulation of their own emotions, as well as in their resilience capacity. The effect size shows that emotional regulation is the most relevant individual variable in terms of differentiating between the two types of aggressors.

Similarly, the results of Table 3 show statistically significant differences between the two types of aggressors in the family variables and relevant effect sizes. Specifically, the generalist aggressors obtained lower scores in secure attachment and higher scores in insecure attachment (preoccupied, avoidant and traumatized). Regarding family functioning, the results also show significant differences between the two types of aggressors. Once again, the generalist aggressors obtained higher scores in parental permissiveness and lower scores in the value granted to parental authority, although the effect size of both variables was very small (*d* < 0.20). The generalist aggressors also presented higher scores in the violence experienced from the parents and in the observation of psychological and verbal violence between the parents. More specifically, the generalists obtained higher scores in all types of parental violence compared to the specialist aggressors. The effect sizes show that violence from the father is the most relevant family variable for differentiating the two types of aggressors.

Finally, with the aim of exploring the predictive capacity of the variables that showed significance in the type of aggressor, logistic regression was performed (see Table 4). The model correctly classified 78.2% of the cases, was statistically significant (χ^2^ (4, 1559) = 173.02, *p* < 0.001, *R*^2^ Nagelkerke = 0.164) and represented approximately 16.4% of the variation in the type of aggressor. Specifically, proactive reasons for the violence towards the mother, reactive reasons for the violence towards the father, low emotional regulation and violence from the father contribute to predicting the generalist type of aggressor.

## 4. Discussion

The present study analyzed, for the first time, the differential profile of specialist aggressor versus generalist aggressor in a community sample of adolescents who had shown violent behavior towards their parents in the last year. In line with similar studies [16,20,25], the distribution found confirms the existence of two types of CPV aggressors. In particular, 22.4% are distributed in the group of specialists, whereas the other 77.6% are distributed in the group of generalists. These percentages are in line with those obtained in a judicial population, where, among 305 adolescent offenders charged with CPV offenses, 14.75% were specialists (only CPV offenses) and 85.25% were generalists (CPV offenses and also other violent (75%) and non-violent offenses (25%)) [13]. As in the cited work, the percentages found in the present study suggest that most CPV occurs within a generalized pattern of violent behavior, indicating the need to pay more attention also to this type of aggressor. Moreover, other types of violence also show a greater proportion of generalists than specialists. For example, Boyle et al. [20] report percentages of 63% and 37%, respectively. Therefore, in line with similar studies, the present work shows, on the one hand, that there are two types of CPV aggressors and, on the other hand, that most of them exert violence both at home and outside the home.

The second objective was to examine the differences between the two types of aggressors in their sociodemographic characteristics and in a set of individual and family variables traditionally linked to CPV, as well as to explore the predictive capacity of the significative variables in the type of aggressor. In line with the findings of similar works [20,25], the present research identified that specialists and generalists differ significantly in all the analyzed variables, except in gender, in control/domain CPV towards the father, in the ability to recognize other people’s emotions and in the observation of physical violence between the parents. Furthermore, the effect sizes reveal that such differences are relevant in many cases, except in parental permissiveness and in the value granted to parental authority. In the specific case of these two variables, the effect size is very small; therefore, these differences must be interpreted with caution, and further research is needed.

Regarding the sociodemographic characteristics, no differences were found in gender between specialists and generalists. These results are not in line with those of Grace-Moulds et al. [13], who obtained a greater proportion of boys among the generalists compared to the specialists. However, given the particularities of the judicial population, it was expected that the results of our community sample were different, and the absence of community studies limits the comparison of our results. Furthermore, the results do seem to suggest a heterogeneous distribution of the aggressors among the different age groups. These data are not in line with those of Grace-Moulds et al. [13], who did not find differences in age, although they are similar to those reported in gender-based violence, where it has been pointed out that generalists are younger than specialists [16]. Nevertheless, our results show an age pattern different from that reported in gender-based violence. Traditionally, adolescence has been studied by differentiating three phases or stages [73]: early (up to 13 years of age), middle (from 14 to 16 years of age) and late adolescence (from 17 years of age onwards). In this study, there seems to be a greater proportion of specialists than generalists in the early and late ages of adolescence (12–13 years and 17–18 years) and a greater proportion of generalists than specialists around the middle ages of this evolutive stage (14–16 years). One possible explanation for our results has to do with opportunity as a necessary element for a person to engage in criminal and violent activities. Criminal opportunities are concentrated in time and can be reduced [74]. In the initial and final ages of adolescence, opportunities to exert generalized violence could be lower, since social contexts are more limited in the case of younger adolescents and more selective in the case of older adolescents. The social circle of the former is usually more limited to the parents, and there are still few contexts for the occurrence of violence towards peers (e.g., street, school, digital media). In the second case, the social circle is usually more selective or restricted since, in general, these adolescents are already integrated in a social group and there is no need to integrate in new groups; therefore, the contexts for the occurrence of violence towards peers are also limited. Social changes produce new criminal opportunities [74]. In line with this, the central stage of adolescence is characterized by a series of more profound changes, such as the expansion of social interactions, the search for group identity and the longing for autonomy and independence, which are aspects that could involve a larger number of social conflicts both inside and outside the family context, with greater opportunities for generalized violence as a way to resolve such conflicts. In any case, further studies should analyze whether age plays a relevant role in specialized and generalized violence in CPV in order to design specific awareness campaigns about both types of violence as a function of age, which would also be useful for the improvement of interventions, adapting them to the evolutionary development of each stage.

The CPV pattern differs between specialists and generalists. Specifically, it was found that the generalist aggressors, in addition to exerting violence in more contexts, showed higher levels of violence towards both their mothers and fathers. As has been reported in similar studies [20,22], this finding suggests that generalists are more violent than specialists, and, moreover, that they are violent towards both parents. Likewise, our results support the study of Grace-Moulds et al. [13], who reported that most generalists show high levels of violence, since 75% of them had committed violent offenses, compared to 25% who had committed non-violent offenses. In addition, our findings provide new data, as it was found that the generalists were more violent towards parents compared to the specialists. Regarding the type of CPV, it was found that the generalist aggressors exerted higher levels of all types of violence than the specialists, except in control/domain CPV towards the father, providing data that had never been analyzed before. Furthermore, in line with the trait-based model of CPV [29], the generalist aggressors exerted more CPV for proactive reasons than the specialists. However, in contrast with the theoretical assumptions of this model, the specialists did not exert more CPV for reactive reasons; on the contrary, it was the generalists who also had more reactive reasons than the specialists. These inconsistencies could be explained by attending to the differences found in this study in the levels of exposure to parental violence [75]. Since the generalists experienced more violence from their parents, they also had more defensive reasons for exerting violence towards their parents, in a possible dynamic of bidirectional violence [60]. More specifically, it was found that the reactive reasons for CPV towards the father and the proactive reasons for CPV towards the mother are the ones that predict the type of generalist aggressor. These results underline the importance of not only including the reasons for CPV in research, but also differentiating them based on the parent towards whom the violence is exerted [36,37,75].

The present study found that specialists and generalists also differed in their individual characteristics. Specifically, the generalists had more difficulties in recognizing, using and regulating their own emotions compared to the specialists, whereas both types recognized other people’s emotions in a similar manner. These results provide empirical support to the trait-based model of CPV, which assumes higher levels of emotional insensibility traits in the generalist aggressors than in the specialist aggressors [29]. Moreover, such results also complement those of previous studies that negatively relate emotional intelligence to CPV [38,40,42] and to violence towards peers [26,44,45], since the greatest deficits of emotional intelligence were found in those adolescents who exert both CPV and violence towards peers. Likewise, low emotional regulation contributes significantly to the prediction of this type of aggressor. Differences were also found between both types of aggressors in resilience capacity. Once again, the generalist aggressors showed greater difficulties in adapting to conflicting and adverse situations. Although resilience has not been thoroughly explored in the literature about aggression, the little evidence suggests that it is negatively related to both CPV and violence towards peers [14,47]. This study expands the understanding of the role of resilience, as it was found that the adolescents who exert these two types of violence are also the ones who show a lower capacity to adapt to conflicting and adverse situations. This finding, together with the previous, suggests that both emotional and coping difficulties may play a relevant role in the explanation of CPV, since a moderate relationship was found between emotional intelligence and resilience [14], and that such difficulties could play a different role in the development of specialized and generalized CPV. Likewise, considering that emotional and coping difficulties were greater in the generalist aggressors than in the specialist aggressors, it suggests the need to work harder on the emotional control and resilience of these aggressors.

This study also explored the family dynamics of these two types of aggressors. Although parental attachment has been previously related to CPV and violence towards peers [14,39,41,46,48], the present study found that the generalists had more insecure emotional bonds with their parents compared to the specialists, being characterized to a greater extent by the preoccupied, avoidant and traumatized attachment styles. In this sense, the aggressors who exert generalized violence are more afraid of being abandoned, experience more rejection, trust their parents less, show more indifference, and experience a greater lack of availability and more violence from their role models [50,51], compared to the aggressors who exert specialized violence. These results provide new data about the role of attachment in CPV. Moreover, it has been highlighted that parental attachment modulates the regulation of oneself and influences relationships with others [70]. In this line, it has been found that some elements of insecure attachment, such as the lack of parental warmth, are related to psychological maladjustments that are, in turn, related to CPV [36]; together with the results of this study, this finding suggests the relevance of this variable in CPV. Furthermore, Belsky [52] stated that the quality of attachment is greatly influenced by the family context. One of the most widely studied variables of the family context is exposure to parental violence. Experiencing violence from the parents or observing violence between the parents has been systematically associated with CPV and also with violence towards peers [6,31,37,43,54,55,56,57,58]. The results of this study show that the aggressors who exert both of these types of juvenile violence experience more parental violence than those who only exert CPV. Specifically, they suffer more psychological, physical and verbal violence from their parents and observe more psychological and verbal violence between the parents. Therefore, the results suggest that higher levels of CPV occur in the type of aggressor that also presents higher levels of parental violence, supporting the conclusions of a recent meta-analysis, which states that minors who have experienced violence from their parents have a 70% higher probability of exerting CPV [76] compared to minors who have not experienced parental violence. It has also been found that father violence, but not mother violence, predicts the type of generalist aggressor, indicating the need to separately analyze the violence exerted and received by the father and the mother [58].

In summary, this study found that there are two types of aggressors in CPV, specialists and generalists, and that they differ in their characteristics. Specialized violence seems to be more frequent at the beginning and end of adolescence, whereas generalized violence appears to be more frequent in the central ages of this life stage. Furthermore, the results show that most CPV aggressors generalize violence towards other people outside the family context, since a large proportion of them also exert violence towards their peers, and that these aggressors show a more negative profile. Specifically, they exert more CPV towards their mothers and their fathers, and for both reactive and proactive reasons. Likewise, they have more difficulties in recognizing, using and regulating their emotions and a lower capacity to cope with conflicts adequately. Regarding the family dynamics, they have more insecure emotional parental bonds and are also more exposed to parental violence, since they suffer more violence from their parents and observe more violence between them. Furthermore, the findings show the need to deepen into the individual and family variables that explain reactive and proactive CPV towards mothers and fathers separately and to analyze possible differential mechanisms with respect to specialist and generalist aggressors.

The study has some limitations that must be taken into account. Firstly, the cross-sectional design does not allow establishing causal relationships. It would be interesting to analyze the temporal sequence of CPV and violence towards peers in future longitudinal studies. Secondly, the results are based on a sample of adolescents from two provinces of southern Spain, limiting their generalization to other geographic and cultural contexts. It is necessary to replicate the results in other types of samples, such as judicial samples, since the profile of both types of aggressors could be different depending on the population studied. Moreover, this study includes those who exert violence towards peers in the generalist category, since this is the type of juvenile violence that has been most widely studied in relation to CPV and to the analyzed variables. However, it would also be interesting for future studies to include other types of violence, such as dating violence, which has also been associated with CPV [34,77], in this category of generalist aggressors. Likewise, although this study analyzed many of the variables of interest in the field of CPV (see the review of Simmons et al. [12]) and in relation to violence towards peers, others were not included. Specifically, future studies could complement our findings by analyzing other variables such as cognitive variables or parental warmth and communication, which could be especially relevant in the category of specialist aggressors [36,37], and mental health or drug use, which could be especially relevant in the category of generalist aggressors [16,17]. Finally, the self-report of the adolescents could be complemented with that of the parents and peers.

## 5. Conclusions

Despite the limitations and although further research is needed, this is the first study to analyze the differential profile of specialist aggressor versus generalist aggressor in CPV. This work provides additional evidence on the efficacy of the dimension of generality of the violence to establish different profiles. It is considered that our findings could have important implications for research. Specifically, this study could be useful for the continuation of the differential analysis of the characteristics of these aggressors. Likewise, the results could help to propose different etiological mechanisms of the development of CPV. Furthermore, although the results allow understanding the pattern of CPV in this profile, they also suggest the convenience of determining the individual and family mechanisms that explain why some adolescents are only violent towards their parents while other adolescents are also violent towards their peers and why the latter show higher levels of CPV. In view of our results, it would be interesting to analyze whether the levels of insecure attachment are linked to emotional and coping difficulties and whether and how these are, in turn, related to specialized and generalized CPV. Moreover, it is considered that our findings could also have important implications for professional practice. Specifically, the study could help to reduce or prevent CPV, as it suggests the need to differentiate both types of aggressors in the design of intervention and prevention programs. Concretely, since a large proportion of generalist aggressors was found, and these are characterized by greater individual and family difficulties, professionals could identify these aggressors with the aim of further training their emotional and coping competencies, working more on the emotional bond with their parents and reducing the levels of intrafamily violence.

In conclusion, we consider that this study provides valuable information in several ways. Firstly, it broadens the analysis of the differential characteristics of both types of aggressors; secondly, it deepens the approach to different etiological mechanisms of the development of CPV; and finally, it assists in the development and implementation of intervention and prevention programs aimed at covering the common and differential needs of specialist and generalist aggressors in CPV.

## Figures and Tables

**Table 1:** Sociodemographic characteristics and differences by type of aggressor.

	Generalists *n* = 1209		Specialists *n* = 349		Total Sample *n* = 1558		$\chi^{2}$	φ
	*n*	%	*n*	%	*n*	%		
Female	663	54.8	188	54.0	851	54.6	0.06	−0.00
Male	547	45.2	160	46.0	707	45.4		
12–13 years	308	25.5	106	30.5	414	26.6	6.9 *	0.07
14–16 years	784	64.8	199	57.2	983	63.1		
17–18 years	117	9.7	43	12.4	160	10.3		

Note: * *p* < 0.05.

**Table 2:** Means, standard deviations and Mann–Whitney *U* test of the pattern of and reasons for child-to-parent violence.

	Generalists *n* = 1210		Specialists *n* = 349		Z	Cohen’s d
	*M*	*SD*	*M*	*SD*		
Violence towards the mother	7.00	5.30	4.35	3.23	−10.04 ***	0.60
Psychological	2.45	2.92	1.03	1.99	−10.36 ***	0.57
Physical	0.23	0.89	0.08	0.43	−3.16 **	0.21
Financial	0.90	1.34	0.35	0.93	−8.31 ***	0.48
Control/domain	3.38	2.37	2.87	1.98	−3.33 **	0.23
Reactive reasons	0.66	0.65	0.39	0.55	−7.82 ***	0.45
Proactive reasons	0.64	0.51	0.36	0.39	−9.67 ***	0.62
Violence towards the father	6.08	5.02	3.88	2.84	−8.37 ***	0.54
Psychological	2.30	2.90	1.02	1.81	−8.92 ***	0.53
Physical	0.21	0.91	0.05	0.24	−3.40 **	0.24
Financial	0.77	1.24	0.32	0.91	−7.31 ***	0.41
Control/domain	2.80	2.26	2.49	1.81	−1.47	0.15
Reactive reasons	0.64	0.65	0.36	0.49	−7.86 ***	0.49
Proactive reasons	0.52	0.49	0.30	0.36	−8.08 ***	0.51

Note: ** *p* < 0.01, *** *p* < 0.001.

**Table 3:** Means, standard deviations and Mann–Whitney *U* test of the individual and family variables.

	Generalists *n* = 1210		Specialists *n* = 349		Z	Cohen’s d
	*M*	*SD*	*M*	*SD*		
Intrapersonal emotional perception	20.03	4.59	21.29	4.76	−4.83 ***	−0.27
Interpersonal emotional perception	21.19	4.09	21.61	3.84	−1.52	−0.10
Emotional assimilation	18.79	5.29	19.91	5.28	−3.70 ***	−0.21
Emotional regulation	15.95	5.47	18.75	5.24	−8.28 ***	−0.52
Resilience	25.60	6.58	27.34	6.41	−4.43 ***	−0.27
Secure attachment	25.28	4.41	26.53	3.78	−5.31 ***	−0.30
Preoccupied attachment	11.30	3.53	10.61	3.42	−3.16 **	0.20
Avoidant attachment	12.32	3.18	11.53	3.20	−4.10 ***	0.25
Traumatized attachment	9.76	3.99	8.55	3.49	−5.42 ***	0.32
Parental permissiveness	7.08	2.26	6.70	2.11	−2.92 **	0.17
Parental authority	12.70	2.03	12.96	1.83	−2.00 *	−0.17
Violence from the mother	2.40	2.75	1.37	2.23	−7.20 ***	0.41
Psychological	0.76	1.07	0.43	0.89	−5.65 ***	0.33
Physical	0.79	1.02	0.46	0.84	−5.49 ***	0.35
Verbal	0.86	1.14	0.47	0.92	−6.03 ***	0.38
Violence from the father	2.64	2.89	1.42	2.28	−8.00 ***	0.47
Psychological	0.84	1.13	0.43	0.88	−6.41 ***	0.40
Physical	0.82	1.03	0.48	0.86	−5.76 ***	0.36
Verbal	0.98	1.18	0.51	0.92	−6.94 ***	0.44
Observation of parental violence	0.80	1.72	0.41	1.04	−3.97 ***	0.27
Psychological	0.51	0.96	0.27	0.71	−4.30 ***	0.28
Physical	0.17	0.59	0.09	0.36	−1.50	0.16
Verbal	0.12	0.46	0.04	0.23	−2.47 *	0.22

Note: * *p* < 0.05, ** *p* < 0.01, *** *p* < 0.001.

**Table 4:** Logistic regression of the study variables in the prediction of the type of aggressor.

	*B*	*SE*	Wald	Exp (*B*)	95% CI
Reasons for child-to-parent violence					
Proactive–mother	1.12	0.17	44.28 ***	3.09	[2.21, 4.31]
Reactive–father	0.43	0.14	9.00 **	1.54	[1.16, 2.04]
Individual characteristics					
Emotional regulation	−0.06	0.01	24.43 ***	0.94	[0.91, 0.96]
Family characteristics					
Violence from the father	0.11	0.03	13.02 ***	1.11	[1.05, 1.18]

Note: Generalist = 1. ** *p* < 0.01, *** *p* < 0.001.

## Data Availability

The data of this research are available upon request to the corresponding author.
